# Combination of intraoperative indocyanine green video-angiography FLOW 800 and computed tomography perfusion to assess the risk of cerebral hyperperfusion syndrome in chronic internal carotid artery occlusion patients after revascularization surgery

**DOI:** 10.3389/fneur.2023.1323626

**Published:** 2023-12-05

**Authors:** Juan Du, Jun Shen, Jian Li, Fayong Zhang, Renling Mao, Yinghua Xu, Yu Duan

**Affiliations:** ^1^Department of Neurology, Huadong Hospital, Fudan University, Shanghai, China; ^2^Department of Neurology, Shanghai East Hospital, Tongji University, Shanghai, China; ^3^Department of Neurosurgery, Huadong Hospital, Fudan University, Shanghai, China; ^4^Department of Neurosurgery, Huashan Hospital, Fudan University, Shanghai, China; ^5^Departments of Anesthesiology, Huadong Hospital, Fudan University, Shanghai, China

**Keywords:** chronic internal carotid artery occlusion, fluorescein angiography, cerebral revascularization, regional cerebral blood flow, hyperperfusion syndrome

## Abstract

**Background and purpose:**

To study the changes of corticocerebral hemodynamics in surgical area and postoperative hyperperfusion syndrome in patients with chronic internal carotid artery occlusion (CICAO) by intraoperative indocyanine green videoangiography (ICGA)-FLOW 800 and CT perfusion after superficial temporal artery (STA)–middle cerebral artery (MCA) bypass surgery.

**Methods:**

From October 2019 to January 2021, 77 patients diagnosed with CICAO underwent direct bypass surgery at Huadong hospital (affiliated with Fudan University) were enrolled. Regions of interest (ROIs) at STA, proximal MCA (PMCA), distal MCA (DMCA), cortical blood capillary (CBC), and cortical vein (CV) were identified after anastomosis by ICGV-FLOW 800 including peak fluorescence intensity (PFI), time to peak (TTP), and area under the time curve (AUC) of fluorescence intensity. All patients underwent perfusion-weighted CT before bypass surgery and those patients with HPS were verified by CTP after bypass.

**Results:**

14 patients with HPS were verified by perfusion-weighted CT after bypass. In HPS group, the AUC_TTP_ of DMCA was significantly larger (T = −3.301, *p* = 0.004) and TTP of CBC was shorter (T = −2.929, *p* = 0.005) than patients in non-HPS group. The larger AUC_TTP_ of DMCA (OR = 3.024, 95%CI 1.390–6.578, *p* = 0.0050) was an independent risk factor by further multivariate logistic regression analysis.

**Conclusion:**

The hemodynamic changes of cortical vessels during STA-MCA bypass surgery could be recorded accurately by ICGV-FLOW 800. Furthermore, the increased AUC_TTP_ of DMCA and shorter TTP of CBC may be potential risk factors of HPS.

## Introduction

Chronic internal carotid artery occlusion (CICAO) is a rare cerebrovascular disease that is characterized by progressive stenosis to occlusion in internal carotid arteries (1). The main treatment for CICAO is revascularization surgery, especially superficial temporal artery (STA)–middle cerebral artery (MCA) bypass surgery, which can directly and efficiently establish sufficient blood flow from the extracranial artery to the intracranial artery to improve cerebral perfusion (2). Hyperperfusion syndrome (HPS) is a common hemodynamics-related complication that may lead to neurological deficits, including cerebral hemorrhage or ischemia, after revascularization (3, 4). However, at present, there are no reliable models and/or methods to predict the occurrence of HPS in patients with CICAO after STA-MCA bypass surgery.

Indocyanine green video-angiography (ICGA) FLOW 800 software (Carl Zeiss Meditec AG) can record time-delayed dynamic video and calculate the hemodynamic parameters of regions of interest (ROIs) at cortical surfaces. ICGA has been gradually introduced in bypass surgery for CICAO because of its high spatial resolution and excellent image quality with reliable real-time parametric variation in cortical vessel flow (5).

In this retrospective cohort study, we investigate the mechanism of HPS in patients with CICAO after STA-MCA bypass surgery and further discuss the determinant by hemodynamic parameters changes in different blood flow periods collected by ICGA-FLOW 800 technology.

## Materials and methods

### Clinical data

From October 2019 to January 2021, patients diagnosed with CICAO were retrospectively enrolled at Huadong Hospital (affiliated with Fudan University). This study was approved by the Ethics Committee of Huadong Hospital (20190030). All patients signed informed consent forms.

Inclusion criteria: aged between 14 and 75 years old; at II–VI period by Suzuki stage with internal carotid artery occlusion or stenosis (6); no prior intracranial and extracranial vascular anastomosis of any kind; received STA-MCA bypass surgery; after anastomosis, and cortex blood flow tested by ICGA. Exclusion criteria: patients with internal carotid artery or MCA occlusion caused by atherosclerosis without a preoperative and/or postoperative computerized tomography perfusion imaging (CTP) test.

### STA-MCA bypass

**Figure 1:**
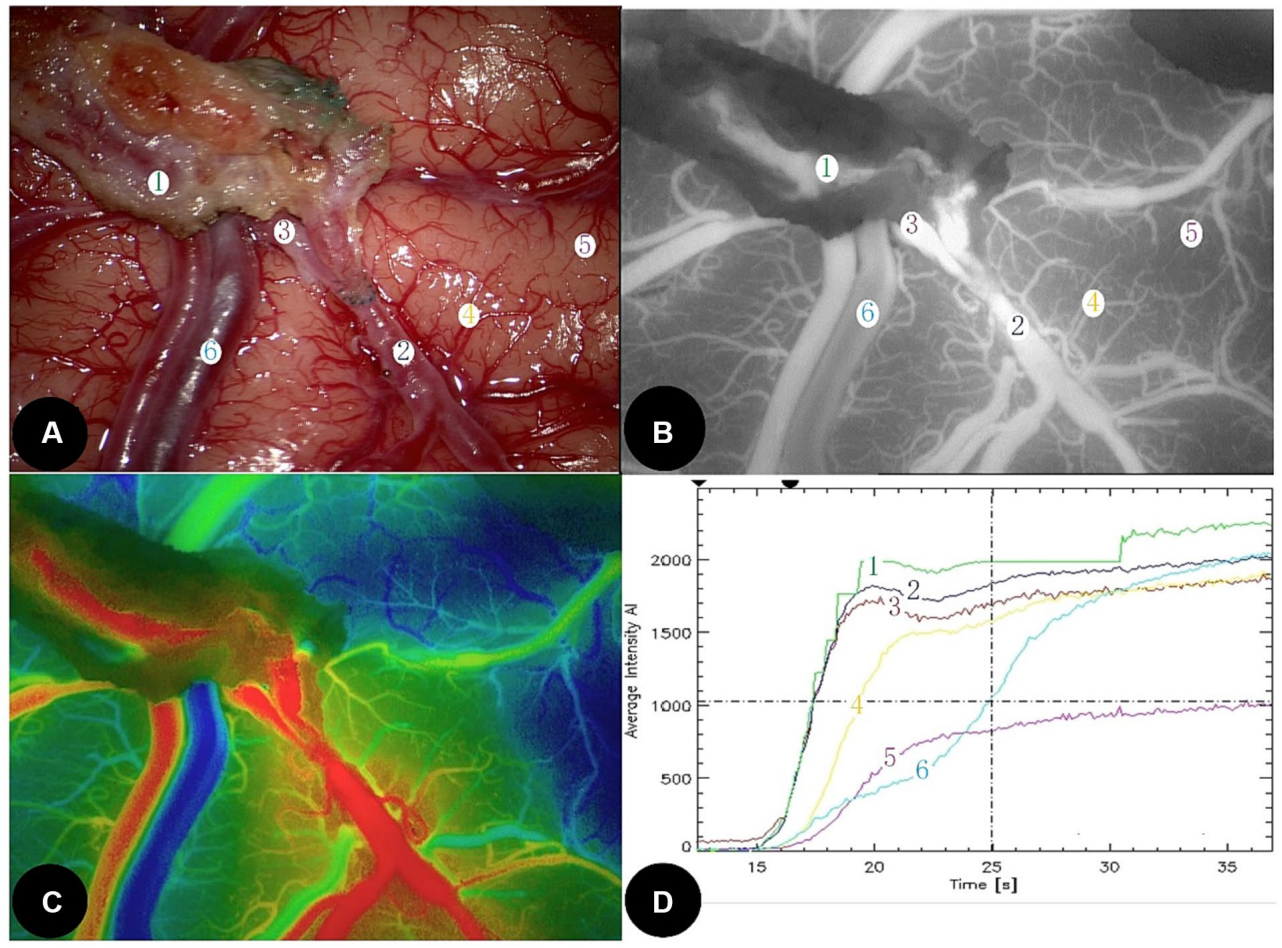
A 56-year-old female developed temporary motor aphasia and dysphagia at 3 days after left STA-MCA direct bypass surgery. **(A)** Microscopic image during bypass surgery. STA was marked as ①, DMCA as ②, PMCA as ③, CBC as ④ and ⑤, and CV as ⑥. **(B)** Intraoperative indocyanine green video-angiography image. **(C)** Schematic diagram of each peak blood flow time; the redder, the peak is the earlier, the bluer, the peak is the later; **(D)** Fluorescence intensity—time curve at each interest vessels. The value of AUC_TTP_ at STA (mark 1) and DMCA (mark 2) increased significantly. STA, Superficial temporal artery; DMCA, The distal part of middle cerebral artery; PMCA, The proximal part of middle cerebral artery; CBC, Cortical blood capillary; and CA, Cortical vein.

To perform bypass surgery, the head of the patient was positioned at 60 degrees to the opposite side. The scalp was then incised, and the temporal muscle was isolated from the temporal lobe. After milling the skull, cutting the dura mater, and confirmation of a suitable receptor (MCA-M4 segment, diameter ≥ 0.8 mm; Figure 1A), STA was towed through the temporal muscle. After anastomosis, 0.2 mg/kg indocyanine green (Dandong Medical and Pharmaceutical Co., Ltd., China) with 10 mL of normal saline was intravenously injected at a high rate of speed. The ROIs at the STA, the proximal part of MCA (PMCA), distal part of MCA (DMCA), cortical blood capillary (CBC), and the cortical vein (CV) around the anastomoses were selected using fluorescence angiography (OPMI Pentero fluorescence microscope, Carl Zeiss, Germany; Figure 1B). If the fluorescence intensity (PFI) of the blood vessel rises early, the vessel will appear darker red in the composite image, whereas if the PFI rises later, the vessel will appear dark blue (Figure 1C). The coordinate plot of the fluorescence intensity over time and its original data were automatically generated using FLOW-800 microscope (Figure 1D). Time to peak (TTP) indicates the time from the beginning of fluorescence to the strongest fluorescence. The area under the curve (AUC) from the origin of the coordinates to the TTP indicated as AUC_TTP_, was calculated using GraphPad Prism 8.0 (GraphPad Software, United States). The value of PFI, TTP, and AUC_TTP_ were analyzed.

### Perioperative management

Maintaining stable blood pressure and ensuring the balance of inflow and outflow, the adverse reactions were recorded in detail. These included the following: (1) persistent headache for more than 72 h; (2) muscle weakness, seizures, and aphasia. Patients with adverse reactions were tested by CT or MRI immediately to exclude intracerebral hemorrhage or a fresh infarction.

### Computerized tomography perfusion imaging acquisition and analysis

**Figure 2:**
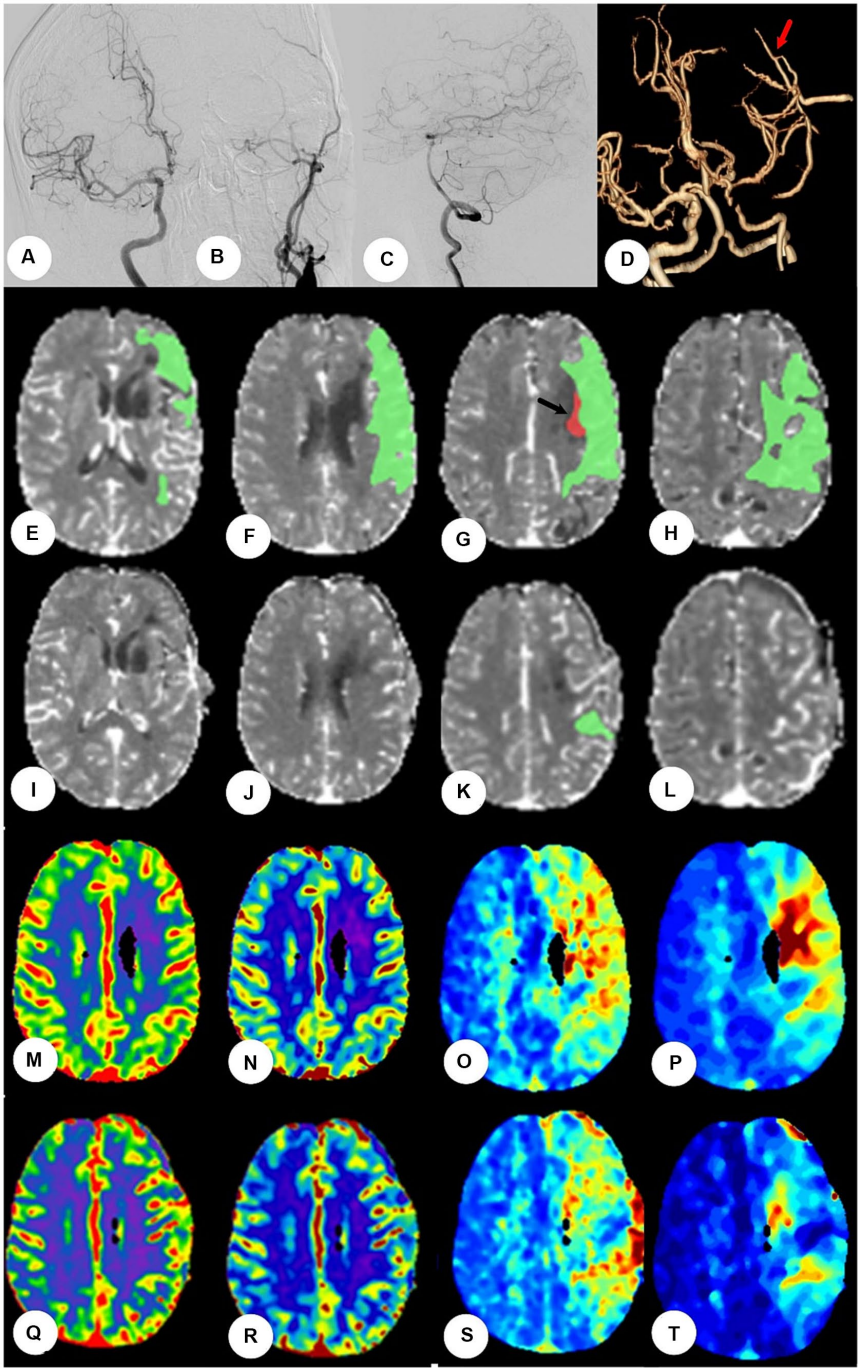
The patients were performed by CTP at the fourth day after bypass surgery. **(A–C)** Digital subtraction angiography showed left initial internal carotid artery was occluded, right the left internal carotid artery and bilateral vertebral artery systems is normal, but without obvious compensation. **(D)** Postoperative CT angiography showed the blood flow at anastomosis was patent (red arrow). **(E–H)**: Preoperative CTP showed the mismatch green area (Tmax>6 s) was 93.1 mL and the hypoperfusion red area (rCBF<30%) was 0.6 mL at frontotemporal parietal lobe (black arrow). **(I–L)** Postoperative CTP showed mismatch area reduced significantly and hypoperfusion area disappeared. **(M–P)** Figures of CTP showed CBV, CBF, and MTT and Tmax, respectively, before bypass. The values of CBV and CBF were normal, the values of MTT and Tmax lengthened at left frontotemporal parietal lobe. **(Q–T)** The values of CBV and CBF were still normal, and the values of MTT and Tmax shortened significantly after bypass surgery.

All patients underwent a CTP test (GE LightSpeed VCT 64 Slice CT Scanner) to determine cerebral perfusion status and not all patients were reviewed for CTP after surgery because of radiation hazards. However, once adverse reaction occurred in patients similar to HPS postoperatively, we would actively review CTP test to determine whether HPS was present. A head retainer was used to keep the patient’s head as stationary as possible during the scan. Ioformol (Hengrui Co. Ltd., China) was administered via the median cubital vein at a flow rate of 5.0 mL/s at a dose of 50 mL. The scan parameters were as follows: tube voltage = 80 kV, tube current = 120–150 mA, layer thickness = 1.25 mm, total scanning time = 60 s; the plateau, inflow, and outflow period were all included. Data were automatically analyzed using brain perfusion CT image processing software (PerfusionGo V1.0; Shukun, Co. Ltd., China) via a singular value decomposition and deconvolution algorithm for dispersion correction (Figure 2).

### Identification of HPS

If patients show postoperative adverse reactions, CTP should be performed. According to a procedure described in a previous study (7), HPS was confirmed by CTP after bypass as follows: (1) Mismatch area and/or hypoperfusion red area (rCBF < 30%) reduce significantly; (2) CBV and CBF increased or MTT and TTP reduced significantly at the anastomotic region; and (3) Without fresh hemorrhage or infarction.

### Statistical analysis

The SPSS v.23.0 statistical software package was used to process the data. Continuous variables were verified using the Kolmogorov–Smirnov normality test. Normal distributional data were represented by mean ± standard deviation ( $\bar{x}$ ±*s*) and the differences between two groups were detected by an independent *t*-test. Nonnormal distributional data were compared using the Mann–Whitney U test. Categorical variables were represented as frequencies and compared by the χ^2^ test. The risk factors for HPS were calculated using multivariate logistic regression. A *p* value of <0.05 was considered statistically significant.

## Results

### General information

A total of 77 patients were included in this study (37 males and 40 females), with an average age of 42.2 ± 13.3 years, ranging from 31 to 72 years. According to the clinical classification criteria of Matsushima, 34 patients suffered from transient ischemic attack (TIA), 24 from cerebral infarction, and 19 from cerebral hemorrhage. According to Suzuki staging, 9 cases were at level II, 21 cases were at level III, 14 cases were at IV level, 19 cases were at level V, and 14 cases were at level VI.

### Postoperative adverse reactions and HPS syndrome

23 patients had moderate to severe adverse reaction after bypass surgery, 20 received postoperative CTP test, and finally, 14 patients (18.2%) were confirmed to be in the HPS group. The main postoperative adverse events were swallowing dysfunction or salivation (five cases), persistent headache (five cases), epilepsy (two cases), and contralateral muscle strength weakness (two cases; Table 1).

**Table 1:** Comparison of clinical data between HPS group and non-HPS group of patients with chronic internal carotid artery occlusion.

Relevant parameters	Univariate analysis				Multiple regression analysis	
	HPS group (*n* = 14)	Non-HPS group (*n* = 63)	Test value	*p* value	OR (95%, confidence intervals)	*p* value
Clinical features						
Male (*n*, %)	6(42.9%)	31(49.2%)	0.185^b^	0.667		
Age (years old, ± s)	50.4 ± 13.3	40.3 ± 13.5	-2.518^a^	0.014		
Hypertension (*n*, %)	3(21.4%)	24(38.1%)	1.397 ^b^	0.085		
Hyperlipidemia (*n*, %)	6(42.9%)	17(27.0%)	1.378 ^b^	0.241		
Diabetes (*n*, %)	4(28.6%)	20(31.7%)	0.054 ^b^	0.817		
Surgery at dominant hemisphere (*n*, %)	11(78.6%)	3(21.4%)	3.584 ^a^	0.058		
Clinical classification			−0.475 ^b^	0.636		
TIA	7(50.0%)	27(42.9%)				
Cerebral infarction	4(28.6%)	20(31.7%)				
Cerebral hemorrhage	3(21.4%)	16(25.4%)				
Suzuki stage			−0.339 ^c^	0.735		
II	1(7.1%)	8(12.7%)				
III	4(28.6%)	17(27.0%)				
IV	2(14.3%)	12(19.0%)				
V	5(35.7%)	14(22.2%)				
VI	2(14.3%)	12(19.0%)				
Systolic blood pressure	108.0 ± 6.3	109.3 ± 5.0	0.792 ^a^	0.431		
Diastolic blood pressure	80.4 ± 6.5	78.9 ± 5.6	−0.921^a^	0.3601		
TTP						
STA	25.45 ± 5.02	27.64 ± 4.58	−1.678	0.093		
PMCA	25.75(23.26,30.48)	27.76(25.37,31.09)	−1.110	0.267		
DMCA	24.74(21.92,30.36)	27.45 ± 4.65	1.356	0.557		
CBC	25.47(22.37,31.95)	29.16(26.23,31.31)	−0.168	0.045	0.892,0.786 ~ 1.012	0.077
CV	28.71(24.06,35.55)	31.73(28.56,34.39)	0.162	0.872		
PFI						
STA	1477.3 ± 110.6	1503.5 ± 79.0	0.149	0.882		
PMCA	1807.4 ± 160.3	1699.7 ± 81.6	−0.57	0.57		
DMCA	2050.5 ± 171.4	1901.0 ± 111.4	−0.597	0.552		
CBC	1613.9 ± 153.1	1467.6 ± 85.3	−0.75	0.455		
CV	1308.1 ± 92.8	1135.7 ± 55.9	−1.361	0.178		
AUC						
STA	1.91 ± 0.79	1.61 ± 0.69	−2.929	0.182		
PMCA	1.84(1.50,2.06)	1.25(0.97,1.56)	−0.205	0.968		
DMCA	2.31(2.01,3.55)	1.84(1.44,2.37)	−3.301	0.004	3.024, 1.390 ~ 6.578	0.005
CBC	1.57(1.37,2.14)	1.39(1.17,1.86)	−1.856	0.012		

^a^*T* value; ^b^*χ*^2^ value; ^c^*Z* value; HPS, Hyperperfusion syndrome.

### Risk factors for HPS syndrome

In the HPS group (14 patients), the AUC_TTP_ of the DMCA was significantly larger (T = −3.301, *p* = 0.004) and the TTP of the CBC was shorter (T = −2.929, *p* = 0.005) compared to that in the non-HPS group. Multivariate logistic regression analysis indicated that the larger AUC_TTP_ of the DMCA (OR = 3.024, 95% CI: 1.390 ~ 6.578, *p* = 0.0050) was an independent risk factor for HPS.

## Discussion

Chronic internal carotid artery occlusion is associated with a 5–8% annual risk of stroke, even under medical treatment (8, 9). STA-MCA artery bypass, as a low-flow bypass, can reduce the risk of recurrence ipsilateral ischemic stroke, however, would result in HPS-significant focal increase in cerebral blood flow at the site of the anastomosis, which is responsible for postoperative neurological deterioration (10). HPS was first reported by Masaaki in 1998 (11) and the occurrence rate of HPS could be from 4.4% to even 67.5% (12–16). In our cohort, the rate was 18.2%, and the HPS-related symptoms included aphasia, hemiparesis, dysarthria, seizure (17–21). A variety of clinical features have been studied as key risk factors associated with HPS, including preoperative hypertension, weak posterior circulation (22), the hemodynamic sources of the recipient parasylvian cortical arteries (23), and a longer delay time at CTP (24). At present, there are still no reliable models to predict the occurrence of HPS in patients with CICAO after bypass surgery.

ICG fluorescence is a new semiquantitative-visual technique that has been applied for the intraoperative detection of cerebrovascular disease (25–27). Zhang et al. further optimized the parameters of ICG mapping to include peak cerebral blood volume, regional cerebral blood flow, and TTP using FLOW 800 software. Moreover, they found that the differences in peak cerebral blood volume and regional cerebral blood flow before and after bypass were significantly higher in the symptomatic group (28).

According to hemodynamic Hagen–Poiseuille equation (29), Hu put forward the basic principle of the ‘flow-controlled’, the hemodynamic difference between the donor and recipient arteries should reduce after STA-MCA anastomosis (30). Compared with the non-HPS group, the forward volume of the STA in the HPS group was significantly lower, identified using a phase-contrast MRI technique in our previous study (20). However, the obvious flaws of phase-contrast MRI technique include excessively long detection times and fuzzy imaging owing to patient movement, which lead to data bias and difficulty in clinical application (20). In operative practice, we believe that high blood flow in the STA after bypass is an important cause of HPS and follow a principle of matching selection of donor and recipient vessels (31). Using the ICG fluorescence angiography technique, we can directly choose ROIs at the cortical surface, including blood vessels, from the arterial to the capillary and venous stage. Its parameters include TTP and AUC, representing blood flow velocity and blood flow volume, respectively, which are more accurate than the parameters employed in previous hemodynamic research techniques.

A higher STA blood flow results in abnormally high local cerebral perfusion. It was verified that after anastomosis, the circulatory resistance of distal STA suddenly and significantly decreased, and the blood flow of the receptor passively increased (32). If the STA undergoes compensatory thickening after bypass, patients will show higher rates of headache (33). In Figures 1A–C, the blood flow of STA was ejected directly into the MCA-M4 receptor at a high rate of speed, and the fluorescence intensity of the STA was the highest at all cortex blood vessels according to automatic analysis of FLOW-800, which showed that the higher blood flow of the STA appeared in the ultra-early period after anastomosis. According to the Mann–Whitney U-pair analysis, the AUI_TTP_ value of the DMCA was significantly larger in the HPS group, confirming that the flow of the DMCA passively increased after bypass.

In contrast, some patients with weaker vascular networks could not withstand changes in blood flow. In this study, the TTP of the CBC was shorter in the HPS group, indicating that the patients with HPS had a weaker buffering function of the CBC than those without. When foreign and reversed STA blood flow is ejected, it may cause further damage to the entire cerebrovascular network, particularly in patients with weaker CBC function. The results of this study proved again that cerebral perfusion around the anastomosis increased after surgery in the HPS group and that if the cerebral perfusion was excessive, postoperative complications could occur (34).

If high HPS risks are found during surgery, several measures will be adopted in our center. Blood pressure should be maintained at <125/85 mmHg by continuous monitoring every 1 h (35). Nimodipine is used routinely in postoperative patients and nicardipine is proposed by intravenous maintenance for those with high blood pressure. Edaravone as a free radical scavenger (36) and Dl-3-n-Butylphthalide approved by Food and Drug Administration of China for treating ischemic stroke (37), are considered to be effective in reducing HPS by our experience. However, the measures for treating HPS also should be assessed by randomized controlled trials for the efficacy and safety.

### Limitation

This study was a single-center, small sample retrospective study, underlying HPS risk factors such as age, comorbidities, and stroke severity were not differentiated, intraoperative anesthesia status was also not considered. Some HPS patients may be missed, because not all patients were tested by postoperative CTP. In the future, prospective randomized controlled trials in multicenter are needed to further confirm its reliability.

## Conclusion

Intraoperative fluorescein videoangiography FLOW 800 could predict the risk of HPS in patients with CICAO after bypass surgery. A large AUC of DMCA is an important risk factor for HPS.

## Data availability statement

The raw data supporting the conclusions of this article will be made available by the authors, without undue reservation.

## Ethics statement

The studies involving humans were approved by Ethics Committee of Huadong Hospital, Fudan University. The studies were conducted in accordance with the local legislation and institutional requirements. The participants provided their written informed consent to participate in this study.

## Author contributions

JD: Writing – original draft. JS: Data curation, Writing – review & editing. JL: Data curation, Writing – original draft, Writing – review & editing. FZ: Funding acquisition, Writing – review & editing. RM: Supervision, Validation, Writing – original draft. YX: Formal Analysis, Methodology, Resources, Writing – review & editing. YD: Conceptualization, Supervision, Writing – original draft.
